# Increasing condom use and declining STI prevalence in high-risk MSM and TGs: evaluation of a large-scale prevention program in Tamil Nadu, India

**DOI:** 10.1186/1471-2458-13-857

**Published:** 2013-09-17

**Authors:** Thilakavathi Subramanian, Lakshmi Ramakrishnan, Santhakumar Aridoss, Prabuddhagopal Goswami, Boopathi Kanguswami, Mathew Shajan, Rajat Adhikary, Girish Kumar Chethrapilly Purushothaman, Senthil Kumar Ramamoorthy, Eswaramurthy Chinnaswamy, Ilaya Bharathy Veeramani, Ramesh Shivram Paranjape

**Affiliations:** 1 National Institute of Epidemiology, ICMR, Chennai, India; 2 FHI 360 India, New Delhi, India; 3 FHI 360 Headquarters, Washington, USA; 4 National AIDS Research Institute, ICMR, Pune, India

## Abstract

**Background:**

This paper presents an evaluation of Avahan, a large scale HIV prevention program that was implemented using peer-mediated strategies, condom distribution and sexually transmitted infection (STI) clinical services among high-risk men who have sex with men (HR-MSM) and male to female transgender persons (TGs) in six high-prevalence state of Tamil Nadu, in southern India.

**Methods:**

Two rounds of large scale cross-sectional bio-behavioural surveys among HR-MSM and TGs and routine program monitoring data were used to assess changes in program coverage, condom use and prevalence of STIs (including HIV) and their association to program exposure.

**Results:**

The Avahan program for HR-MSM and TGs in Tamil Nadu was significantly scaled up and contacts by peer educators reached 77 percent of the estimated denominator by the end of the program’s fourth year. Exposure to the program increased between the two rounds of surveys for both HR-MSM (from 66 percent to 90 percent; AOR = 4.6; p < 0.001) and TGs (from 74.5 percent to 83 percent; AOR = 1.82; p < 0.06). There was an increase in consistent condom use by HR-MSM with their regular male partners (from 33 percent to 46 percent; AOR = 1.9; p < 0.01). Last time condom use with paying male partners (up from 81 percent to 94 percent; AOR = 3.6; p < 0.001) also showed an increase. Among TGs, the increase in condom use with casual male partners (18 percent to 52 percent; AOR = 1.8; p < 0.27) was not significant, and last time condom use declined significantly with paying male partners (93 percent to 80 percent; AOR = 0.32; p < 0.015). Syphilis declined significantly among both HR-MSM (14.3 percent to 6.8 percent; AOR = 0.37; p < 0.001) and TGs (16.6 percent to 4.2 percent; AOR = 0.34; p < 0.012), while change in HIV prevalence was not found to be significant for HR-MSM (9.7 percent to 10.9 percent) and TGs (12 percent to 9.8 percent). For both groups, change in condom use with commercial and non-commercial partners was found to be strongly linked with exposure to the Avahan program.

**Conclusion:**

The Avahan program for HR-MSM and TGs in Tamil Nadu achieved a high coverage, resulting in improved condom use by HR-MSM with their regular and commercial male partners. Declining STI prevalence and stable HIV prevalence reflect the positive effects of the prevention strategy. Outcomes from the program logic model indiacte the effectiveness of the program for HR-MSM and TGs in Tamil Nadu.

## Background

Men who have sex with men (MSM) and transgender persons (TG) are among the most vulnerable core-risk groups affected by HIV. Five to 10 percent of the global HIV prevalence is reportedly attributed to sexual transmission involving MSM [1]. In India, among the estimated 2.5 million people living with HIV (PLHIV) [2], the main mode of HIV transmission is sexual contact, both heterosexual and homosexual [3]. While the contribution of male-to-male sex to the overall HIV epidemic is not known, the large population of sexually active MSM (estimated to be 2.35 million) [4] and high rates of HIV prevalence in this population are of considerable concern. According to the National AIDS Control Organisation (NACO), India’s nodal agency for HIV/AIDS prevention and control, HIV prevalence among MSM in India was estimated to be 7.4 percent in 2009 [5], much higher than the 4.9 percent reported for female sex workers (FSWs) [6]. Higher prevalence rates among MSM have also been reported by other studies in such major cities as Chennai (9 percent) and Mumbai (11–18.9 percent) [7-9]. HIV Sentinel Surveillance data also suggests increasing HIV prevalence among MSM during 2003–2007 in Tamil Nadu (4.2–6.6 percent) and Karnataka (10.8–17.6 percent) [10].

India is recognized as having multiple concentrated HIV epidemics [11], hence it is important to address the most-at-risk groups like MSM and TGs, who may be fueling the spread of HIV through high-risk sexual practices [7,8]. Many studies have documented high-risk behaviors of MSM, who often have many sex partners, also have sex with women [12,13], report low rates of condom use [12] and also indulge in other risky behaviors such as drug use [14]. All of these risks make MSM highly vulnerable to HIV and other sexually transmitted infections (STIs). Notably, studies have also shown that HIV prevalence in male-to-female TGs is significantly higher than in MSM [8]. Indeed, it has been argued that TGs are a separate group socially and economically, and may have unique health needs [15]. Thus, currently there is an emphasis on separating TGs from other MSM in health programs.

NACO has implemented targeted HIV prevention interventions among FSWs, MSM and TG since 1992 [4]. In 2003, the Bill & Melinda Gates Foundation initiated Avahan: the India AIDS Initiative, a large-scale program aimed at checking the spread of HIV in India by focusing on key high-risk groups [16]. Avahan was implemented in 83 districts across the six Indian states most affected by the epidemic. The initiative’s main objective was to rapidly deliver large-scale prevention intervention and prevent further transmission of HIV. Its services were primarily directed at MSM and TGs who were 18 years or over, the most visible and located in high-risk sex venues or cruising sites. The program therefore classifies the MSM population targeted by Avahan as high-risk MSM (HR-MSM) and TGs (locally referred to as *aravani* in Tamil Nadu). The initiative’s key strategies for reaching its goals were to saturate HIV-prevention program coverage among HR-MSM and TGs (target of 80 percent) and deliver a proven package of services [17,18] to address the determinants of HIV risk in these populations [19].

One of Avahan’s six focus states was the southern state of Tamil Nadu, where the program covered FSW, HR-MSM, and TG populations across 14 of the state’s 32 districts between October 2004–March 2009. Key elements of the Avahan program for HR-MSM and TGs in Tamil Nadu included peer-based outreach and education for behavior change, promotion and distribution of condoms, STI treatment services, and community mobilization [17].

A comprehensive adequacy and plausibility evaluation was designed to assess the Avahan program’s scale, coverage, outcomes and impact sequentially over time and described elsewhere [20]. With this framework, based on the program’s logic model, and using monitoring data and independent survey data, the present study evaluated the impact of the Avahan program on HR-MSM and TGs in Tamil Nadu. The specific objectives of the evaluation were: examine the coverage and scale achieved by the program for HR-MSM and TGs, assess changes in selected program outcomes, and identify any links between these outcomes and exposure to the program. The present analysis also describes findings on changes in HIV and STI prevalence, reported condom use among HR-MSM and TGs, and gaps that call for programmatic attention.

## Methods

### Data sources

Data for the present analysis came from three main sources:

(i) Routine program monitoring data on outreach and clinic services [21,22] from the 14 Avahan implementation districts of Tamil Nadu provided information on coverage. Table 1 provides a list of the program monitoring indicators that were examined.

(ii) Two rounds of cross-sectional integrated behavioral and biological assessment (IBBA) surveys were conducted separately for HR-MSM and TGs in 2006 (Round 1) and 2009 (Round 2). The surveys collected socio-demographic and behavioral data in face-to-face interviews and took blood and urine samples to test for STIs and HIV. The operational definition of the HR-MSM surveyed by IBBA was same as the Avahan program — male, aged 18 years or older, visible in cruising sites or sex venues and who had had anal sex with another man in the last one month in exchange for cash or kind. In both rounds of IBBA, HR-MSM were sampled in four districts (Chennai, Coimbatore, Madurai and Salem) using time location cluster sampling, a probability-based sampling method. TGs were sampled in five districts (Chennai, Coimbatore, Dharmapuri-Krishnagiri, Salem and Madurai) and operationally defined as any individual who self-identified as aravani, aged 18 years or above, who had any type of sex (oral, manual, penetrative), paid or unpaid, with another male in the last one month. Since HR-MSM and TGs are highly mobile and sex venues/cruising sites keep changing, mapping exercises were carried out separately for each group in both rounds of surveys to develop a comprehensive sampling frame. Two-stage sampling protocol was used: in the first stage, primary sampling units (PSU) were selected using probability proportion to size (PPS) from the sampling frame; this was followed by the second stage, where a random selection of respondents was done based on listing at selected clusters. Time location cluster sampling was used to sample HR-MSM and TGs from street-based sites such as markets and bus stops; conventional cluster sampling was used for TGs when respondents were from fixed sites such as homes. Further details of IBBA survey protocol and the methodology have been published elsewhere [22].

(iii) Another set of data came from condom distribution by Avahan program and sales from Avahan-supported and other social marketing efforts [23,24].

**Table 1:** Framework for evaluation

**Evaluation question**	**Indicator**	**Data source**
**1. Is coverage of Avahan adequate?**	**A. Scale**	
	a. ***Geographical coverage-*** Description of rollout in number of districts and change in number of implementing NGOs over time	Central monitoring information system (CMIS)
	b. ***Proportion of HR-MSM ever contacted and ever visited clinic-*** Number of HR-MSM ever contacted by Avahan peer educators or ever visited Avahan program STI clinics divided by the estimated size of HR-MSM as of March 2009	CMIS
	c. **Proportion of HR-MSM c*ontacted monthly by peer educators or visited program STI clinics for STI consultations –*** Number of HR-MSM contacted every month by peer educators or visited program STI clinics monthly, divided by the estimated denominator/size of HR-MSM as of March 2009	CMIS
	d. ***Proportion of HR-MSM/TGs contacted in last month-*** Percentage of HR-MSM from IBBA who reported that they had been contacted by Avahan peer educators in the month preceding survey	IBBA
	**B. Intensity**	
	a. ***Number of peer educator/outreach worker and ratio of MSM to peer educators-*** The total number of active outreach workers and peer educators in the Avahan intervention areas across implementation districts in Tamil Nadu; number of estimated MSM/TG covered per peer educator in the coverage area	CMIS
	b. ***Condom distribution and availability***	CMIS and condom social marketing data (CSM)
	1. Absolute number of free condoms distributed by the Avahan program annually and condom sales from project-supported condom social marketing by program from 2005 to 2008.	
	2. ***Condom needs analysis-*** Ratio of average monthly condoms available per MSM; total condoms distributed by Avahan and made available through project-supported condom social marketing sales, divided by the estimated number of MSM in the area covered by Avahan; and ratio of number of condoms distributed to monthly commercial sex acts per MSM/TG, where sex acts are calculated based on number of sex acts with paying and paid male partners per month multiplied by total estimated number of MSMs covered by Avahan multiplied by four to get monthly sex acts***.	
	3. Proportion of HR-MSM reporting source of obtaining condom last time from outreach worker/peer educator/nongovernmental organization	IBBA
	c. ***Frequency of contact by peers HR-*** MSM/TG reporting number of times they were contacted by peer educators in the month preceding the survey	IBBA
	d. ***Frequency of visit to clinic HR-*** MSM reporting the number of times they visited the Avahan program clinics for STI services	Individual level CMIS data
**2. Has there been an increase in condom use by HR-MSM?**	**Change in condom use pattern**	
	a. Proportion of HR-MSM reporting last time condom use with paying male partners during two rounds of IBBA	IBBA
	b. Proportion of HR-MSM reporting consistent condom use with paid male partners during two rounds of IBBA	IBBA
	c. Proportion of HR-MSM reporting consistent condom use with regular male partners during two rounds of IBBA	IBBA
	d. Proportion of HR-MSM reporting consistent condom use with other non-commercial male partners during two rounds of IBBA	IBBA
**3. Has there been a reduction in STIs and new HIV infections?**	**Change in STI prevalence and visits to clinic with STI symptoms**	
	a. STI prevalence (reactive syphilis serology, high-titre syphilis, gonorrhoea (NG), chlamydia (CT), any STI (NG, or CT or high-titre syphilis)	IBBA
	**Change in HIV prevalence and new HIV infections**	IBBA
	a. HIV prevalence among HR-MSM aggregated from all districts in two rounds of IBBA	
	c. HIV prevalence among HR-MSM in the age group of 18–20 years	
**4. Is Avahan exposure associated with increase in condom use and declining STIs?**	**Association of intermediate outcomes and STIs to program exposure**	IBBA
	a. Program exposures, defined as exposure to any one — ever contacted by peer, ever visited program clinic, and ever received condoms from peer educators; its link to consistent condom use with commercial and non-commercial partners, using pooled data from two rounds of IBBA	
	b. Duration of program exposure and its link to condom use with commercial and non-commercial partners, using pooled data from two rounds of IBBA	
	b. Program exposure, as defined above, and its link with presence of any STIs (NG, CT or high-titre syphilis)	

*** In both rounds of IBBA, HR-MSM were asked about the number of times they had anal sex with a paying male partner in the past one week; for paid male partners, it was the number of times they had anal sex in past one month.

### Analytical framework for evaluation

The current analysis was conducted using data from the 14 Avahan implementation districts in the state of Tamil Nadu, for the period of January 2005–March 2009. The analytical framework is similar to Avahan’s evaluation design [18]. The framework addresses four questions to assess the following: 1) scale and intensity of Avahan coverage among HR-MSM and TGs in Tamil Nadu; 2) changes in condom use; 3) changes in the prevalence of STIs and HIV; and 4) possible links between intermediate outcomes and exposure to the Avahan program.

### Indicators

#### Program coverage and intensity

Coverage indicators [22] from monitoring data included: ever contacted by peer educators, ever visited program STI clinics and ever received condoms. Avahan’s target for coverage (80 percent of the estimated denominator in Avahan districts) was the benchmark against which the adequacy of achieved coverage was compared. The estimated size of the HR-MSM/TG population in Avahan districts, as of March 2009, was used for this analysis (see Table 1). Service indicators were examined against Avahan’s established targets for outreach (one contact per month) and clinic visits (once per quarter, or 33 percent of the estimated denominator for HR-MSM/TGs per month).

Measures of program intensity included: ratio of the program staff (peers and outreach workers) to target group (against a target of 1:50 HR-MSM and TGs) and the frequency of exposure to Avahan program services. The number of free condoms provide by the program and sold through program-supported social marketing efforts [22] were used to assess gaps in condom availability. Clinic monitoring information system (MIS) data provided details on the frequency of visits to Avahan clinics over time [25].

IBBA data on self-reported exposure (evaluated coverage) to Avahan services was analyzed and used for comparing and validating the program monitoring data.

### Intermediate outcomes

#### Condom use

IBBA collected self-reported condom use data to examine changes in condom use patterns. Consistent condom use was defined as reported condom use during every act of anal intercourse in the past with any male sexual partner, including regular male partners, paid male partners (when buying sex) and other casual male partners; last time condom used captured information on condom use with paying male partners (when selling sex).

#### Sexually transmitted infections (including HIV)

Blood samples were tested for syphilis using rapid plasma reagin (RPR) and a confirmatory *Treponema pallidum* hemagglutination assay (TPHA). Positive RPR confirmation by TPHA was used to define reactive syphilis or lifetime syphilis. RPR titres of ≥1:8 with a confirmatory TPHA were defined as active or high-titre syphilis. Urine was tested using nucleic acid amplification (Gen-Probe APTIMA COMBO 2) to assess chlamydial and gonococcal urethral infection [26]. Rectal specimens could not be collected due to practical considerations, difficulty of collecting these samples in the field setting and the survey implementation protocol.

Blood samples were also tested for HIV infection with a two-test algorithm using an enzyme immunoassay (J. Mitra, New Delhi) [26]. HIV prevalence among young MSM (18–20 years) was examined as a proxy for new HIV infections.

### Association between outcomes and program exposure

Data from the two rounds of IBBA surveys was used to study the link between exposure to Avahan services and the presence of any STIs (urethral gonorrhoea, chlamydia or high-titre syphilis) and the practice of consistent condom use with commercial and non-commercial partners. A composite indicator of exposure — ever been exposed to any of the three core program services (contacted by peer educator, visited program STI clinics and received condoms from peers) — was used for this analysis (see Table 1). Dose response was assessed by examining, from both IBBA Round 1 and Round 2 data, the duration of exposure to Avahan program and consistent condom use.

### Statistical analyses

Survey data were double entered using CSPro Software (U.S. Census Bureau, Washington, DC). SPSS 14.0® (IBM, Somers, NY) was used to analyze the data. District level data were merged to get state data set from each round. Pooled IBBA Round 1 and 2 dataset was used to analyse the association between condom use and exposure. Appropriate district- or state-level weights were calculated and used for analysis [26].

The Wald Chi-square test was used to assess significant changes in profile characteristics of HR-MSM and TGs between the two IBBA rounds, including age, literacy, marital status, income source, age at first sex, residency, sex work outside the place of residence and sexual self-identity.

Separate multivariate logistic regression was conducted to assess: a) significant changes over time in exposure; b) condom use outcomes with each partner type; c) changes in prevalence of STIs and HIV between the two rounds; and d) the link between exposure to Avahan services and intermediate outcomes. The key profile variables associated with outcomes — self-identity, age, literacy, occupation, residency, age at first sex for HR-MSM; and self-identification as aqua (non-castrated) or nirvana (castrated) aravani, district, age, literacy, occupation, age at first sex and residency for TGs — were controlled for confounding in logistic regression models. Adjusted odds ratios (AORs) and 95 percent confidence intervals (95% CI) were generated. Chi-square test was used to examine the association between condom use and duration of Avahan exposure using pooled IBBA data. The association was considered significant for p-values lower than 0.05.

### Ethics statement

Protection of Human Subjects Committee of FHI 360 and the ethics committee of National Institute of Epidemiology (NIE), Indian Council of Medical Research (ICMR), approved the study.

## Results

### Program coverage and intensity

The Avahan program for HR-MSM and TGs in Tamil Nadu was initially rolled out in 14 districts. By April 2007, it was stabilized in 11 districts through 24 non-governmental organizations (NGOs) working in these districts. During 2007–2008, one implementation district was transferred from Avahan to NACO and another district from NACO to Avahan, based on the state plan for implementing phase III of India’s National AIDS Control Plan [4]. By March 2009, the estimated size of HR-MSM and TGs served by the program in these 11 districts stood at 15,100.

#### Coverage data from MIS

**Figure 1:**
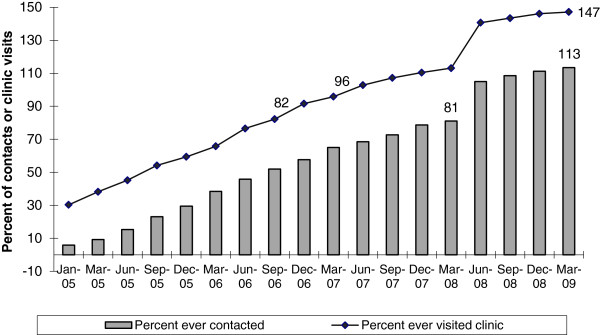
**Percent of HR MSM/ TGs in Avahan districts in Tamil Nadu who were ever contacted and ever visited program STI clinics - Avahan CMIS 2005–2009.** This figure shows the increase in proportion of HR MSM / TG who were ever contacted peer Avahan program peer educators (given in bars) and proportion who had ever visited the program STI clinics (given as a line graph) over the period 2005–2009 when Avahan was implemented.

HR-MSM and TGs ever contacted by Avahan peer educators increased to 82 percent (12,412) of the estimated HR-MSM and TG population by September 2006, while those who had ever visited program STI clinics increased to 81 percent (12,237) by March 2008. By March 2009, these percentages had increased to 147 percent and 113 percent, respectively (see Figure 1).

**Figure 2:**
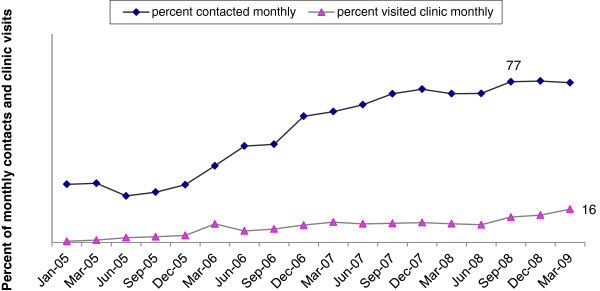
**Percentage of HR MSM / TG in Avahan districts of Tamil Nadu who were contacted monthly and who visited STI clinics monthly- Avahan CMIS 2005–2009.** This graph shows the consistent increase in the monthly coverage / utilization by HR MSM/ TG over time between 2005 and 2009 through Avahan implementation.

Monthly contacts by Avahan peer educators increased consistently from 28 percent in the three months of program inception to 77 percent by March 2009. However, number of HR-MSM and TGs making monthly visits to Avahan STI clinics remained low, 6 percent and 8 percent between 2006 and 2008 respectively, and increased to 16 percent by March 2009 (see Figure 2).

**Figure 3:**
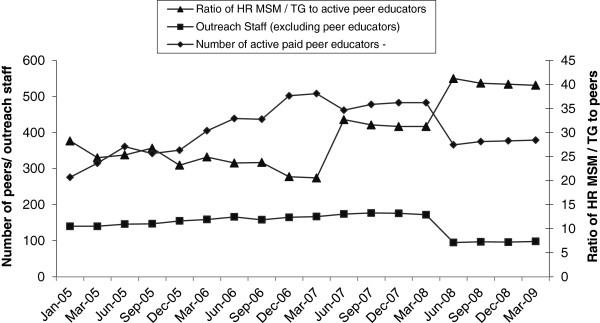
**Active Peer educators / Outreach workers and Ratio of HR MSM/ TG to peer educators from Avahan Districts in Tamil Nadu- Avahan CMIS 2005–2009.** This graph shows that the ratio of peers / and outreach workers to the target population of HR MSM / TG was maintained with required adjustments (as per change in population size) throughout the duration of the Avahan program implementation.

The number of peer educators increased consistently from March 2007 and stabilized by March 2009 (see Figure 3). The number of outreach workers also increased through March 2008, but declined thereafter. The ratio of HR-MSM to peer educators increased from one peer for every 30 MSM in 2005 to one peer for every 40 MSM by March 2009 (see Figure 3).

#### Clinic MIS

**Figure 4:**
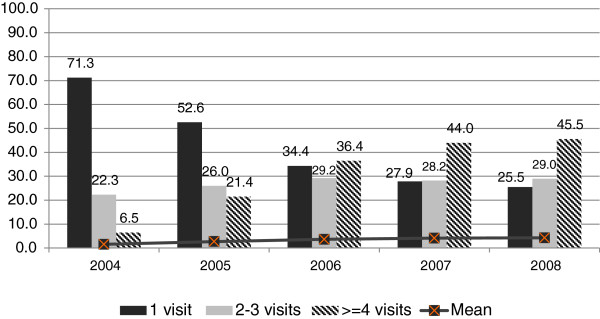
**Frequency of visits by HR MSM to Avahan STI clinic in Tamil Nadu, 2004–2008.** This graph shows the increasing frequency of visits made by HR MSM to Avahan STI clinics in the state, with increasing frequency of visits over time (between 2004 and 2008).

Clinic MIS data shows increased frequency of visits by HR-MSM to the Avahan STI clinics in the state. The proportion of HR-MSM who visited clinics more than once in the previous year increased from 29 percent in 2004 to 74 percent in 2008; the proportion of those who visited four or more times in the year increased from 6.5 percent in 2004 to 46 percent in 2008 (see Figure 4).

#### Coverage data from IBBA

A total of 3,241 HR-MSM (1,621 in Round 1 and 1,620 in Round 2) and 807 TGs (404 in Round 1 and 403 in Round 2) were sampled. Significant differences were seen in the profile characteristics of HR-MSM between Round 1 and Round 2, including in age, literacy, occupation, residency in the district, age at first sex, occupation, and self-identity (see Table 2). A significantly higher proportion of TGs identified as not castrated (or aqua aravani) in Round 2 than in Round 1 (58.5 percent vs. 37.4 percent; p < 0.001). Additional socio-demographic and sex work characteristics of HR-MSM and TGs are presented in Table 2.

**Table 2:** Socio-demographic and sex work characteristics of HR-MSM and TGs in Round 1 and Round 2 of IBBA

		**HR-MSM**			**Transgender**		
**Characteristics**	**Sub-groups**	**Round 1**	**Round 2**	**Wald-Pearson p-value**	**Round 1**	**Round 2**	**Wald-Pearson p-value**
		**(%; n = 1621)**	**(%; n = 1620)**		**(%; n = 404)**	**(%; n = 403)**	
Self-identity	Kothi	54.3	80.1	<0.01			
	Panthi	11.9	1.3				
	Double decker	31.2	14.5				
	Bisexual	2.7	4.0				
	Aqua aravani				37.4	58.5	0.00
	Nirvana aravani				62.6	41.5	
Current age (years)	< 25	39.44	27.1	<0.05	33.6	26.9	
	25-29	24.9	30.4		29.5	30.9	0.18
	30-34	14.2	15.9		16.3	24.1	
	35-39	9.1	12.8			11.3	
	40+	12.4	13.8		11.2%	6.9	
	Mean	29	29.2		28.8	28.7	
Literacy	Cannot read or write	21.9	8.3	<0.01	34.9	11.2	0.00
Marital status	Currently married	23.8	24.7	0.05	20.1	21.3	
	Ever married	.79	.68		4.8	1.5	
	Never married	74.8	74.6		75.2	77.1	0.14
Main income	Unemployed/student	8.2	3.8	<0.01	30.6	2.6	0.00
	Self-employed/business	17.9	29.3		10.0	9.7	
	Laborer	32.5	38.7		15.7	12.5	
	Service (govt./pvt.)	32.4	17.2		5.1	9.5	
	Sex work	4.4	5.3		20.2	41.0	
	Others (transport workers)	4.3	5.6		18.4	24.7	
Residency	Lives in same city as survey	65.6	98.7	<0.01	94.7	99.6	0.00
Age at first sex (years)	< 15	21.6	37.5	<0.01	52.6	48.1	0.41
	15+	78.4	62.5		47.4	51.9	
Sex work outside residence district	Yes	45.3	18.9	<0.01	39.3	40.5	0.83

The proportion of HR-MSM who reported ever being contacted by a peer educator from the Avahan program increased significantly from 66 percent in Round 1 to 90 percent in Round 2 (AOR = 4.6; 95% CI: 3.1–6.8; p < 0.001). HR-MSM who reported ever visiting Avahan program’s STI clinics increased significantly from 67 percent to 82 percent (AOR = 1.8; 95% CI: 1.3–2.7; p < 0.001). A significantly higher proportion of TGs reported that they were contacted by a peer educator from the Avahan program in Round 2 compared to Round 1 (83 percent vs. 74 percent; AOR = 2.0, 95% CI: 1.1–3.7; p = 0.03). However, the proportion of TGs attending STI clinic reduced significantly in Round 2 compared to Round 1 (44 percent vs. 75 percent; AOR = 0.5, 95% CI: 0.3–0.9; p = 0.02).

The proportion of HR-MSM who reported during IBBA surveys that they were contacted by peer educators in the last month increased significantly from 64 percent in Round 1 to 90 percent in Round 2 (AOR = 5.2; 95% CI: 3.4–7.8; p < 0.001). The reported frequency of contact by peers increased between the two IBBA rounds — the percentage contacted 1–2 times increased from 46 percent in Round 1 to 57 percent in Round 2, and those contacted 3–4 times increased from 28 percent in Round 1 to 38 percent in Round 2 (p < 0.001). The proportion of TGs who reported being contacted by a peer educator in the last month reduced in Round 2 compared to Round 1, but was not statistically significant (91 percent vs. 96 percent; AOR = 0.5, 95% CI: 0.2–1.4; p = 0.16).

#### Condom availability

Avahan’s condom distribution initiative for all target groups (FSWs, HR-MSM and TGs) in Tamil Nadu saw a sharp increase, from about 800,000 in 2004 to 6.1 million in 2008. Similarly, the volume of condoms distributed through social marketing outlets in Avahan districts increased from more than 400,000 in 2004 to about 3 million in 2008. The monthly ratio of condoms distributed by the program directly to HR-MSM and TGs and through social marketing efforts increased from 6 per HR-MSM and TGs in 2005 to 26 per HR-MSM in 2008. The estimated number of monthly commercial sex acts per MSM, based on Round 2 IBBA data, was 15 in 2009. IBBA data also showed that a significantly higher proportion of HR-MSM in Round 2 (87 percent), compared to Round 1 (55 percent), reported receiving condoms on the last occasion from peers educators or NGOs (p < 0.001); this increase was also reported by the TG population (82 percent in Round 2 vs. 74 percent in Round 1).

### Intermediate outcomes

#### Changes in condom use

Condom use with regular male partners improved considerably over the two rounds of IBBA (see Table 3). The HR-MSM reporting consistent condom use with these partners increased from 33 percent to 46 percent (AOR = 1.9; 95% CI: 1.3–2.7; p < 0.01). About 94 percent HR-MSM in Round 2 reported condom use during last sex with a paying partner (when selling sex), compared to 81 percent in Round 1 (AOR = 3.6; 95% CI: 2.2–5.9; p < 0.001). Consistent condom use with paid male partners increased over the two rounds of the survey, but not significantly. However, consistent condom use with other male casual partners increased significantly, from 27 percent in Round 1 to 55 percent in Round 2 (AOR = 4.2; 95% CI: 2.6–6.7; p < 0.01). Notably, consistent condom use with regular female partners was low — 1.5 percent to 8 percent in Round 2.

**Table 3:** Multivariate analysis of condom-related outcomes among HR-MSM and TGs in Round 1 and Round 2 of IBBA

**Condom-related outcomes and STI prevalence**	**Round 1**	**Round 2**	**Crude OR**	**Adjusted OR^**	**p-value**
	**% (N)**	**% (N)**	**(95% CI)**	**(95% CI)**	**(Wald test) Adjusted OR**
**3.1 Condom use indicators for HR-MSM**					
Last time condom use with regular male partner	72.6 (1265)	83.2 (1276)	1.8 (1.3-2.7)	1.9 (1.3-2.9)	<0.01
Last time condom use with male paying partner	80.8 (927)	93.6 (1410)	3.5 (2.2-5.6)	3.6 (2.2-5.9)	<0.01
Last time condom use with paid male partner	66.3 (364)	94.2 (215)	8.3 (2.2-30.8)	11.2 (1.3-101.9)	<0.05
Last time condom use with casual male partner	73.9 (949)	92.5 (883)	4.3 (2.4 – 7.9)	5.2 (2.9-9.1)	<0.01
Consistent condom use with regular male partner	32.9 (1265)	46.3 (1276)	1.8 (1.2-2.5)	1.9 (1.3-2.7)	<0.01
Consistent condom use with paid male partner	42.1	55.2	1.7 (0.82-3.5)	1.9 (0.90-4.4)	0.14
Consistent condom use with casual male partner	26.6 (949)	55.1 (883)	3.4 (2.0-5.6)	4.2 (2.6-6.7)	<0.01
**3.2 Condom use indicators for TGs**					
Last time condom use with regular male partner	73.3 (268)	60.9 (279)	0.56 (0.3-0.9)	0.59 (0.3-1.1)	0.12
Last time condom use with paying male partner	93.1 (297)	79.5 (359)	0.28 (0.13-0.63)	0.36 (0.2- 0.8)	0.02
Last time condom use with other casual male partner	80.7 (105)	67.3 (167)	0.49 (0.2-1.2)	0.25 (0.8-0.8)	0.02
Consistent condom use with regular male partner	34.5 (268)	47.2 (279)	1.7 (0.9-2.9)	1.37 (0.7-2.7)	0.35
Consistent condom use with other casual male partner	18.0 (105)	51.5 (167)	4.8 (1.8 – 13.2)	1.89 (0.6-5.9)	0.27

*^Multivariate models were controlled for the following variables for HR-MSM analysis:* self-identity, age, literacy, occupation, residency, age at first sex*; and the following variables for TG:* self-identification as an aqua or nirvana aravani, district, age, literacy, occupation, age at first sex, and residency.

Legend: Table 3 provides proportion of HR-MSM and TGs using condoms with different partners in R 1 and R 2, along with the crude and adjusted odd ratios from multivariate analysis.

Among TGs, consistent condom use with the regular male partner increased from 34.5 percent in Round 1 to 47.2 percent in Round 2, but was not statistically significant (AOR = 1.37; 95% CI: 0.70–2.69; p = 0.35). TGs reported an increase in consistent condom use with casual male partners, up from 18 percent in Round 1 to 51.5 percent in Round 2, but this increase was not statistically significant (AOR = 1.89; 95% CI: 0.61–5.91; p = 0.27). Table 3 provides data on consistent condom use and last time condom use with different types of male partners.

#### Changes in STIs and HIV prevalence

Syphilis prevalence declined significantly from 14.3 percent in Round 1 to 6.8 percent in Round 2 (AOR = 0.37; 95% CI: 0.23–0.59). Prevalence of urethral gonorrhoea and Chlamydia, less than 1 percent in both Round 1 and Round 2, did not change significantly between the two rounds (see Table 4). Prevalence of HIV increased between Round 1 (9.7 percent) and Round 2 (10.9 percent), but the change was not significant (p = 0.57).

**Table 4:** HIV, syphilis and STI prevalence among HR-MSM and TGs in Tamil Nadu in Round 1 and Round 2

	**Round 1**	**Round 2**	**Crude OR**	**Adjusted OR^**	**p-value**
	**% N = 2032**	**% N = 2006**	**(95% CI)**	**(95% CI)**	**(Wald test)**
**HR-MSM**					
HIV-1 infection	9.7	10.9	1.2 (0.7-1.9)	1.1 (0.7-1.9)	0.57
Reactive RPR + positive TPHA	14.3	6.8	0.43 (0.3-0.7)	0.37 (0.2-0.6)	<0.01
Reactive RPR >1:8 + positive TPHA	3.63	3.43	0.94 (0.5-1.8)	1.15 (0.6-2.35)	0.68
Chlamydia infection	0.68	0.51	0.74 (0.3-2.1)	2.2 (.5-8.9)	0.29
Gonorrhoea infection	0.07	0.15	2.0 (0.2-18.3)	6.9 (0.3-159.5)	0.23
Chlamydia and/or gonorrhoea infection	0.76	0.56	0.75 (0.3-2.2)	2.3 (.6-9.4)	0.24
***HIV prevalence (district-wise)***					
Chennai	4.8	10.9	2.5 (1.1-5.2)	2.6 (0.97-6.8)	0.06
Coimbatore	6.5	11.2	1.8 (0.94-3.5)	2.2 (0.97-5.0)	0.06
Madurai	22.3	14.4	0.59 (0.24-1.4)	0.57 (0.23-1.4)	0.23
Salem	5.5	4.8	0.87 (0.29-2.6)	0.56 (0.18-1.7)	0.31
***Syphilis prevalence (district wise)***					
Chennai	12.9	9.9	0.74 (5.0-16.6)	0.62 (0.3-1.4)	0.24
Coimbatore	14.5	6.3	0.39 (0.2-0.7)	0.37 (0.2-0.8)	<0.01
Madurai	17.8	6.6	0.34 (0.1-1.2)	0.25 (0.1-0.9)	<0.05
Salem	12.2	1.9	0.14 (0.1-0.4)	0.08 (0.0-0.2)	<0.01
**TGs**					
HIV-1 infection	12	9.8	0.79 (0.4-1.6)	1.3 (0.5-3.2)	0.57
Reactive RPR + positive TPHA	16.6	4.2	0.22(0.1-0.4)	0.30 (0.1-0.7)	0.01
Reactive RPR >1:8 + positive TPHA	0	0	-	-	-
Chlamydia infection	0	0	-	--	-
Gonorrhoea infection	0	0	-	-	-

*^Multivariate models were controlled for the following variables for HR-MSM analysis:* self-identity, age, literacy, occupation, residency, age at first sex*; and the following variables for TG:* self-identification as aqua or nirvana aravani, district, age, literacy, occupation, age at first sex, and residency.

Legend: Table 4 provides estimates of HIV prevalence and STI prevalence among HR-MSM and TGs; estimates for HR-MSM provided are overall and district-wise for HIV and syphilis; adjusted odds ratios are provided for change in estimates between IBBA R 1 and R 2.

Syphilis declined significantly in all districts except Chennai, where the decline was not found to be significant in logistic regression analysis. HIV prevalence in Chennai increased significantly; whereas in other districts no significant difference was found between the two rounds in a logistic regression analysis (see Table 4). Prevalence of HIV among young HR-MSM (18–20 years old) increased from 2.1 percent in Round 1 to 4.2 percent in Round 2, but the change was not significant (p = 0.478).

HIV prevalence in TGs reduced from 12 percent in Round 1 to 9.8 percent in Round 2; this reduction was, however, not statistically significant. Similarly, sero-positivity for syphilis in TGs reduced from 16.6 percent in Round 1 to 4.2 percent in Round 2, a statistically significant reduction (AOR = 0.30; 95% CI: 0.13–0.66; p = 0.003). No TGs tested positive for chlamydia or gonorrhoea in either of the two IBBA rounds.

### Association between outcomes and program exposure

Using logistic regression, we found a significant and positive association between exposure to program services and condom use with different partners (see Table 5). Consistent condom use with regular male partners, condom use during last sex with a paying partner, and consistent condom use with other casual male partners were all significantly linked with exposure to any Avahan program services. In the HR-MSM exposed to the program, condom use with all partners increased significantly with increasing duration of exposure (<12 months to >33 months) to the program (see Table 6). No link was found between program exposure and the prevalence of STIs.

**Table 5:** Association of Avahan program exposure to condom use outcomes and STI presence among HR-MSM and TGs in Tamil Nadu

**Condom use**	**Group**	**% who received any services**	**Crude OR (95% CI)**	**Adjusted OR^ (95% CI)**	**p-value (Wald test)**
**Last time condom use with paying male partner**	**HR-MSM**	**88.3**	**5.3** (3.4-8.3)	**4.78** (2.9-7.9)	**<0.01**
	**TGs**	**86.8**	1.6 (0.8-3.0)	2.82 (1.3-6.1)	**<0.01**
**Consistent condom use with regular male partner**	**HR-MSM**	**90.7**	**3.46** (2.3-5.2)	**3.98** (2.3-6.9)	**<0.001**
	**TGs**	**43.1**	1.74 (0.9-3.4)	1.66 (0.8-3.3)	**0.14**
**Consistent condom use with paid male partner**	**HR-MSM**	**74.8**	**0.76** (0.5-1.3)	**1.96** (0.9-4.4)	**0.10**
**Consistent condom use with other male partner**	**HR-MSM**	**93.1**	**5.46** (3.4-8.9)	**5.18** (2.9-9.2)	**<0.01**
	**TGs**	**38.8**	4.2 (1.6-11.1)	5.46 (1.7-17.4)	**<0.01**
**STI prevalence**					
**Any STIs (NG, CT or high-titre syphilis)**	**HR-MSM**	**3.95**	**0.70 (0.34-1.4)**	**0.66** (0.4-1.1)	**0.138**
**Syphilis**	**TGs**	**11.2**	**1.6 (0.8-3.3)**	**1.6 (0.7-3.8)**	**0.25**

^FOR HR-MSM - Multivariate models were controlled for the following variables: age, literacy, marital status, occupation, self-identity, age at first sex, and residency; FOR TGs - Multivariate models were controlled for the following variables: age, literacy, marital status, occupation, self-identity, age at first sex, residency, and district.

Legend: Table 5 provides results of multivariate analysis of the link between condom use and presence of any STIs with Avahan program exposure; for both HR-MSM and TGs, using IBBA data pooled for R 1 and R 2.

**Table 6:** Duration of exposure to program services and reported condom use by HR-MSM in Tamil Nadu (using IBBA R 1 and R 2 pooled data)

	**Duration of program exposure**					**p-value (Wald)**
	**Not exposed (%)**	**<12 months**	**12.23 months**	**24-33 months**	**>33 months**	
**Last time condom use with regular male partner**	13.4	8.6	20.6	26.8	29.6	<0.01
**Last time condom use with male paying partner**	9.9	8.3	16.9	27.3	36.2	<0.01
**Last time condom use with paid male partner**	27.8	9.5	11.3	23.6	27.3	<0.05
**Last time condom use with other casual male partner**	13.9	8.9	22.5	25.8	28.5	<0.01
**Consistent condom use with regular male partner**	8.9	7.3	15.7	27.9	37.4	<0.01
**Consistent condom use with other casual male partner**	5.9	7.5	14.4	30.6	40.8	<0.01

Legend: Table 6 provides proportion of the HR-MSM using condoms with different partners against the duration of Avahan program exposure, using IBBA R 1 and R 2 pooled data.

In the TG population, we found that those exposed to the Avahan program were more likely to report condom use during the last sex act with a paying male partner compared to those not exposed to the Avahan intervention (AOR = 2.82, 95% CI: 1.31–6.1; p < 0.01). Similarly, TGs exposed to the intervention were significantly more likely to report consistent condom use with other male partners than those not exposed to the intervention (AOR = 5.46, 95% CI: 1.72–17.37; p < 0.01).

## Discussion

This paper presents one of the first systematic evaluations of a large-scale prevention program for HR-MSM and TGs in Tamil Nadu. An adequacy and plausibility evaluation design [27] was used to evaluate data from Avahan program monitoring, large-scale surveys and social marketing of condoms. The findings provide strong evidence that the Avahan program achieved high coverage of HR-MSM and TGs, increased program intensity over time, and was linked to increased condom use by HR-MSM with their commercial and non-commercial partners.

According to program monitoring data, the percentage of HR-MSM and TGs who were ever contacted by a peer educator or had ever visited a clinic increased over time to over 100 percent, by March 2009. Unique monthly contacts (against the estimated denominator) by Avahan peer educators increased to 77 percent by March 2009, close to the program’s saturated coverage target of 80 percent. IBBA data from a subset of districts showed that by 2009, 90 percent of HR-MSM and TGs had been contacted in the previous month. These IBBA findings validate the monitoring data and bolster confidence in monitoring data for the districts that were not covered in IBBA.

A plausible explanation for the increase beyond 100 percent for those who were ever contacted and had ever visited a clinic is the turnover in the HR-MSM and TG population, that is, some target population who had accessed the services had moved out and were not counted as part of the denominator in March 2009. MSM and TG populations are known to be mobile, so it is likely that some who had received the services moved out of Avahan coverage areas over time.

Examination of scale-up and intensity measures showed that the targets established for program infrastructure (peers and outreach workers) were in place by June 2007. The observed reduction in the number of active peers in March 2007 and June 2008 was likely due to revised program strategies [17] and the transition of some districts from Avahan to the state as part of phase III of the national program.

Increasing frequency of HR-MSM who reported being contacted by peer’s points to the increasing uptake of program services. Continuous quality monitoring assessments of Avahan STI clinics (reported annually) have also shown consistent improvement in the quality of STI clinical management, operations and performance [28,29].

Analysis of condom availability data indicates that the program distributed a large number of condoms; this was validated by the IBBA data. The analysis also indicated that the minimum number of condoms required to cover the estimated commercial sex acts by HR-MSM had been made available by March 2008. Other studies also provide evidence that a sufficient number of condoms were made available through social marketing; more than 80 percent hot spots in Avahan districts were found to have a greater number of condom distribution outlets [23], and condom procurement and use among bridge groups was 79 percent in 2008 [24]. The state Behavioral Surveillance Study (BSS) in 2009 also put the voluntary condom procurement by MSM across the state at 88 percent in 2009 [30].

IBBA data on HR-MSM profile characteristics showed considerable change over the two rounds of IBBA, with a substantial increase in the proportion of HR-MSM who reported their self-identity as a *kothi* (MSM who are generally receptive during anal sex) [31]. This change may have been an indirect result of the intervention, in that the increasing intensity of intervention allows MSM to ‘come out’ and gain an increased understanding of their sexual orientation and self-identity. The sampling method used may have also contributed to these profile differences. Since PPS sampling was used following the mapping exercises in both IBBA rounds, different PSUs/sites could have been selected during the sampling in each round. This is an inherent limitation of a cross-sectional survey using PPS methodology, which helped provide a representative sample at the district level. Further, the sampling frame universe developed during each round of the IBBA survey represents a universe of HR-MSM and TGs in each surveyed district, which is impacted by population changes and turnover.

Significant increases were seen in condom use with the regular male partner, paying male partners (when selling sex) and casual male partners. Evaluation of HR-MSM programs with a similar package of services in other Asian countries has shown increasing condom use with commercial and non-commercial partners after program implementation [32]. A meta-analysis of interventions for MSM, mainly in western countries, found that behavioral interventions significantly reduced unprotected anal sex by 23% among MSM, similar to results found in our study [33].

Although consistent condom use increased between the two rounds, many high-risk sexual practices with both commercial and non-commercial partners were still being reported. In addition, reported consistent condom use with paid partners did not increase, suggesting the need for greater programmatic effort. Additional studies and analyses are required to better understand the factors affecting condom use patterns among MSM with multiple/different partners, so as to inform programs in developing more effective behavior change communication. The finding that condom use outcomes were linked to Avahan program exposure is strongly suggestive of the program’s effectiveness. However, causality could not be determined with the current analysis, given the lack of a proper control group and the limitations of cross-sectional data.

Our evaluation showed that the prevalence of HIV stabilized (9.7 percent to 10.9 percent) and the prevalence of syphilis declined (14.3 percent to 6.3 percent) in the HR-MSM in IBBA districts. Data prior to Avahan is available only from small studies in select locations. For example, in Chennai, a study of 774 men reported HIV prevalence of 6.5 percent and prevalence of any laboratory-diagnosed STI at 22 percent among HR-MSM [13]. Another study conducted in 2008, using non-probability-based methods, across 18 cities in Tamil Nadu, reported 9 percent HIV prevalence and 8 percent syphilis prevalence [9], quite similar to our IBBA findings.

Increased HIV prevalence in HR-MSM of ages 18–20 years, though not statistically significant, is still an issue of concern and requires in-depth examination. In some IBBA districts, such as Chennai and Coimbatore, increase in HIV prevalence despite increased program exposure is a cause of concern. The program in Chennai was transitioned to the Tamil Nadu state in 2008, but Coimbatore continued to be an Avahan intervention district throughout the study period. Given that consistent condom use in general remained low in HR-MSM, inconsistent condom use could likely have been a major contributor to HIV transmission, resulting in our finding that overall HIV prevalence did not change between the two rounds of IBBA.

Male-to-female TGs formed an important part of our interventions. Interestingly, we found similar HIV prevalence in TGs and other MSM. These findings are in contrast with findings from other parts of the country. Indeed, studies from Mumbai and Pune (in western India) consistently reported HIV prevalence in TGs to be significantly higher than MSM [8,34,35]. Setia and colleagues found that HIV prevalence in TGs was 68 percent, compared to 17 percent in MSM. Similarly, Sahasrabuddhe and coworkers reported HIV prevalence among TGs at 45 percent, compared to 19 percent in MSM [35]. As reported elsewhere [35], TGs were more likely than MSM to report sex work as a main source of income; in fact, this was reported by a majority of TGs in our sample. Although consistent condom use increased with regular and casual male partners over the two rounds of IBBA, condom use during last sex act with a paying male partner and casual male partner showed a reduction. Thus, while sex work was reported by a higher number of TGs, there was simultaneous reduction in condom use with paying partners, putting TGs at a higher risk for HIV and STI transmission. As stated earlier, TGs are a separate socio-economic group from other MSM; thus, public health programs should be encouraged to have separate interventions specifically designed for this group.

This assessment is among the first to show evidence of declining STI prevalence in MSM in Tamil Nadu after the implementation of a large-scale prevention program. Although the finding is encouraging, one of its limitations is that only urethral STIs were examined in this assessment. Given the difficulties in field implementation, it was not possible to collect samples for assessing rectal STIs, which other studies have shown to be high among HR-MSM [36]. There was found to be no link between decline in STIs and program exposure; however the prevalence of STIs in Tamil Nadu was low, therefore the analysis of exposed versus non-exposed was based on small numbers of cases and likely limited by lack of statistical power.

While the sentinel surveillance data has limitations of a small sample size and potential bias due to non-representative recruitment methods, data from two sites (250 sample from each) in Tamil Nadu notably indicated HIV prevalence at 6.8 percent in 2004, which increased to 7.6 percent in 2007 and then declined to 3.6 percent in 2008 [37,38]. However, sentinel surveillance data on HIV prevalence among male attendees of STI clinics suggests that HIV prevalence among men in general has been increasing in many districts [37,38].

The major limitations of cross-sectional studies apply to the current analysis as well. While the sampled population was representative of the HR-MSM and TG populations in each round of IBBA, changes in the universe of these populations must be accepted. Further, the denominators used to assess coverage also have limitations. Estimates of HR-MSM and TG denominators were based on formal mapping exercises by Avahan NGOs. However, size estimates varied in terms of rigor and frequency of updation, with size estimation information in some areas being updated only once every one or two years.

One key limitation of the current evaluation is the lack of proper baseline data and control groups. Round 1 of IBBA was conducted in 2006, about 14 months after the Avahan program was implemented in the state. Due to ethical considerations, control groups were not included in evaluation design. Therefore, it is impossible to definitively attribute outcomes to the program. Although some other programs have ongoing HR-MSM and TG interventions in other districts of Tamil Nadu, data from these programs is not available for comparison. Since Avahan was the sole intervention in the IBBA districts (except Chennai) and high levels of coverage was achieved (based on program size estimates), sub-analysis of IBBA data from HR-MSM and TGs who were not exposed to Avahan program had limited power because of small numbers.

Notably, in a majority of Avahan districts (except Chennai) Avahan’s was the first and only intervention for HR-MSM and TGs. Given this, our findings have provided early evidence of the effectiveness of Avahan program for HR-MSM in Tamil Nadu. However, further analysis and studies are required to better understand the factors that affect the condom use patterns of HR-MSM and TGs with their different partners. Understanding these factors would help HIV prevention programs develop more effective behavior change communication strategies.

The practical considerations discussed earlier, the mobile nature of HR-MSM and TGs, diffusion effect of the intervention, and the program’s intent to rapidly scale-up and transition to existing government programs made it difficult to have any control groups [18] for the evaluation. Therefore in the current evaluation, we used the accepted approaches for evaluating large-scale public health programs [39-42]. The strength of our analysis is that it provides early evidence of the effectiveness of Avahan program for HR-MSM and TGs according to the program logical framework, providing ‘congruency’ of expected trends [27].

## Conclusion

The current evaluation study provides evidence of the successful implementation of the Avahan program for HR-MSM in Tamil Nadu. Using the program logic model, the study shows consistent scale up of the program and high levels of service uptake by HR-MSM and TGs; this was validated by independent IBBA survey data. The HIV prevalence rate did not change much, but syphilis prevalence in HR-MSM and TGs showed a decline. Although further gains in condom use are urgently required, exposure to Avahan program services was linked with condom use, which is a strong indication of the program’s effectiveness.

## Competing interest

The authors declare that they have no competing interest.

## Authors’ contributions

TS and LR contributed to the conception, design, writing and finalization of the manuscript. SA, SR and EC helped with acquisition and interpretation of data and finalization of the manuscript. GP and BK contributed through concept development, data analysis and final editing of the manuscript. GCP’s contribution lay in acquisition of biological data, interpretation of data and editing of the manuscript. MS and IV also contributed to data analysis and interpretation of data. AR and PR contributed in concept development and finalization of the manuscript. All authors read and approved the final manuscript.

## Pre-publication history

The pre-publication history for this paper can be accessed here:

http://www.biomedcentral.com/1471-2458/13/857/prepub

## Data sources

IBBA data are available to outside investigators through application. See website: http://www.nari-icmr.res.in/.
